# Moderators’ Experiences of the Safety and Effectiveness of Patient Engagement in an Asthma Online Health Community: Exploratory Qualitative Interview Study

**DOI:** 10.2196/58167

**Published:** 2025-04-25

**Authors:** Helen E Wood, Georgios Dimitrios Karampatakis, Neil S Coulson, Nishanth Sastry, Xiancheng Li, Stephanie J C Taylor, Chris J Griffiths, Anna De Simoni

**Affiliations:** 1 Wolfson Institute of Population Health Barts and The London School of Medicine and Dentistry Queen Mary University of London London United Kingdom; 2 Centre for Applied Respiratory Research Innovation and Impact Nuffield Department of Primary Health Care Sciences University of Oxford Oxford United Kingdom; 3 School of Medicine University of Nottingham Nottingham United Kingdom; 4 Surrey Centre for Cyber Security Department of Computer Science University of Surrey Guildford, Surrey United Kingdom; 5 School of Business and Management Queen Mary University of London London United Kingdom; 6 Nuffield Department of Primary Health Care Sciences University of Oxford Oxford United Kingdom

**Keywords:** asthma, online health communities, moderators, peer support, digital health, qualitative research, patient safety

## Abstract

**Background:**

Among 5.4 million people receiving treatment for asthma in the United Kingdom, more than 2 million experience suboptimal control, leading to the use of health care services and resulting costs as well as poorer quality of life. Online health communities (OHCs) are increasingly used as a source of lay health advice, providing opportunities for learning and mutual support and complementing information from “official” health sources. While engagement with OHCs has the potential to improve self-management, concerns remain about the reliability and usefulness of the information posted. Professional moderation of such communities is essential for supporting sensitive patients, ensuring adherence to forum guidelines, and maintaining clinical safety.

**Objective:**

This study aims to examine the experiences of moderators in an asthma OHC, identifying challenges and possible areas to optimize the safety and effectiveness of patient engagement.

**Methods:**

All 6 current moderators of a nationwide charity-hosted OHC participated in in-depth, semistructured, audio-recorded, remote interviews. Audio recordings were transcribed verbatim and qualitatively analyzed using reflexive inductive thematic analysis.

**Results:**

The 6 moderators interviewed comprised 4 (67%) specialist respiratory nurses, 1 (17%) volunteer patient ambassador, and 1 (17%) customer support manager (all female, with average age 45, SD 10.5 y). In total, 5 (83%) moderators had at least a year’s experience of OHC moderation. Three main themes were generated from data analysis: moderation processes, challenges to effective moderation, and OHC effectiveness. The first theme focused on the different moderator roles and tasks undertaken, including the application of OHC guidelines in dealing with inappropriate content. The second theme covered difficult issues, such as mental health, and practical challenges, including lack of time and concerns about missing problematic posts. The third theme focused on the factors that made the OHC effective and increased its effectiveness, including keeping users safe, generating more OHC activity, encouraging discussion, and raising awareness of the OHC. We found a contradiction in how the moderators perceived the OHC’s effectiveness and their role in moderating it. While they expressed concerns about having insufficient time to moderate the OHC, they also felt that it was underused and would be more effective if it were busier or more active.

**Conclusions:**

Building on the challenges experienced by the moderators, several recommendations were put forward to optimize the safety and effectiveness of the asthma OHC. Moderators often work in isolation without external training or interaction with others. More research into OHC moderation is needed. A continuous professional development framework could improve moderation quality and user support, aligning with the evolving needs of these communities. These results can be relevant to national and international policy, attempting to enhance the safety of patients’ engagement with OHCs.

## Introduction

### Background

Although 5.4 million people are receiving treatment for asthma in the United Kingdom [1], an estimated 2.17 million have uncontrolled asthma, meaning a greater disruption in everyday activities and a higher risk of an asthma attack [2]. Poor asthma control has a major impact on the use of health care services, accounting for 2% to 3% of the primary care consultations, 60,000 hospital admissions, and 200,000 bed days per year in the United Kingdom [1]. Asthma costs the United Kingdom about £1.1 billion (US $1.4 billion) per year from health care costs and disability claims [3]. Optimizing the effectiveness of online health communities (OHCs) is an important goal, given the recent recommendation that peer support and learning via “digital therapeutic communities” is needed to complement National Health Service online resources [4]. OHCs are increasingly used as a source of lay health advice from peers (a peer is defined as someone being of equal standing or belonging to the same social group based on age, grade, or status, in this case, being someone with the same or similar health condition or concern [5]). Among people with a long-term condition who are internet users, 1 in 4 go on the web to find others with similar health concerns [6].

In the United Kingdom in 2020, 92% of the population reported recent internet use [7], and 63% of the internet users reported seeking health information online [8]. With millions of people potentially accessing information and advice posted by peers in OHCs, this raises pressing questions about the efficacy and safety of this new “informal” medium and how to realize and maximize its untapped potential for health benefit. Our regular discussions with patient and public involvement groups have reaffirmed not only the benefits of online peer support, including improved self-management and shared decision-making with clinicians, but also the risks and difficulties, such as reliability and usefulness of information and anxiety about being judged by the community [9-11]. Peer support is defined as “a system of giving and receiving help founded on key principles of respect, shared responsibility, and mutual agreement of what is helpful...It is about understanding another’s situation empathically through...shared experience” [12].

### Prior Work

Few studies of OHCs have focused on the role of moderators in OHCs [13-18]. The role of health professional moderators in OHCs involves responsibilities, duties, and limitations that they may perceive as difficulties in providing users with adequate online medical service. Being a health professional moderator can be personally and professionally “(dis)empowering” [19]. Moderation within OHCs is not limited to ensuring content quality but is far more complex—providing supportive spaces for disclosure of personal stories and enabling individuals to freely express their perspectives while making sure the content of posts is not causing harm to other users. This is especially relevant in, for example, mental health OHCs [20]. The important role of patient and peer moderators has also been highlighted [17,18], particularly the different and complementary expertise that they bring to the role, resulting from personal experience of a certain health condition [15].

The Asthma + Lung UK (ALUK) asthma OHC is one of over 300 public OHCs, covering over 250 health condition areas, hosted by HealthUnlocked. Initially set up by the charity Asthma UK (before it merged with the British Lung Foundation to become ALUK), the asynchronous text-based OHC has over 23,000 members and over 25,000 posts [21]. There are approximately 2000 active users per month; that is, not all users are active all the time.

Superusers—a select minority group of highly active OHC users found to be essential to the successful spread of self-management information and support and in keeping the forum cohesive over time—act, at times, as moderators of OHC discussions [22]. However, appointed moderators are distinct from superusers. Research has shown that compassionate moderators provide important support for patient engagement in OHCs. Moderators maintain community guidelines, help answer questions, and provide medical expertise [13]. OHCs are often moderated by appointed staff from the hosting platform, with some being additionally moderated by health care professionals. Successful moderation in OHCs requires a multitude of practices to manage the challenges that arise in these communities, gained either via specific training [17] or through experience [13]. Some practices are implemented as preventive measures, such as setting community guidelines and offering coaching to members, while other practices require active intervention, such as providing information and welcoming new members [17]. In general, moderators are received positively by community members and do not appear to interfere with peer interactions [14].

### Goal of This Study

In this study, we sought to further explore the role of moderators within OHCs and specifically examine their experiences with the ALUK asthma OHC. The aim was to identify challenges and potential areas where users’ safety and the effectiveness of OHC engagement could be optimized.

## Methods

### Study Design

We followed a qualitative, one-to-one, semistructured interview design to enable an in-depth exploration of the experiences of the moderators of the ALUK asthma forum.

### Recruitment

We invited all currently active moderators (6 as of November 2022) to participate in the study. Moderators were initially contacted by HealthUnlocked via email on behalf of the research team. The email included an invitation letter, a participant information sheet (Multimedia Appendix 1) providing details of the study and research team, and an informed consent form (Multimedia Appendix 2). The invitation letter asked potential participants to contact a member of the research team (GDK) via email. Once an expression of interest was received, GDK replied to potential participants and arranged a mutually convenient time for the interview. GDK also sent a reminder email the day before the scheduled interview date. The researchers had no contact with the participants before they participated in this study.

### Data Collection

Participation was voluntary. GDK and HEW conducted the interviews remotely between November 2022 and December 2022 using the Zoom (Zoom Communications, Inc) platform. Interviews were conducted during normal office hours at a time convenient to the participant. We did not ask participants to specify where they were during the interview or whether anyone else was present in the vicinity, although we did not observe anyone else besides the participants. The interviews, which lasted 30 to 60 minutes, were concluded when participants confirmed that they had nothing further to add. GDK is a male postdoctoral research associate with experience in qualitative research, and HEW is a female experienced postdoctoral researcher and research project manager with training in qualitative research. At the start of the interview, after introductions to establish mutual rapport, we gave participants an opportunity to ask questions and asked them to reconfirm their consent for the interview to be recorded. Interviewers followed a topic guide composed of open-ended questions and prompts to explore the moderation process (Multimedia Appendix 3), including additional topics that arose during an interview and were later added to the topic guide for subsequent interviews. Any notes that we took during interviews were used as an aide-mémoire and were not analyzed. We audio recorded interviews using the record function within Zoom Cloud (video recordings were not retained).

### Data Analysis

Audio recordings were transcribed verbatim by a professional transcribing agency and checked for accuracy by HEW. We analyzed the transcripts using reflexive thematic analysis according to the 6 steps described by Braun and Clarke [23] (data familiarization, data coding, identifying themes, re-examining themes, defining and naming themes, and synthesizing the report). Reflexivity was ensured by the fact that we neither had experience moderating an OHC or any kind of online health group nor using OHCs. HEW coded the data inductively in Microsoft Word and Microsoft Excel, ascribing a single code to each different idea, and then refined and verified the coding by collating data assigned to the same code, sorting it into categories, and discussing all stages of the process with GDK. We then re-examined and collapsed the categories into possible themes with associated subthemes, following detailed discussions with the whole research team. We did not discuss data saturation as there was no option to interview further participants. Inductive coding enabled categories and themes to emerge naturally from the data, thereby enabling the representation of the participants’ unique perspectives. We neither provided participants with a transcript of their interviews nor asked for their feedback on either the transcripts or findings.

As one of the moderators (the customer support manager) mentioned posts being automatically flagged by the system used on the HealthUnlocked platform, we sought clarification regarding this process via a debriefing meeting to give context to the data from the moderator interviews.

### Ethical Considerations

This study was reviewed by the Queen Mary University of London Institutional Research Ethics Committee, with approval granted on August 18, 2022 (QMERC22.279). We obtained written informed consent from participants before their interview using a web-based consent form (Multimedia Appendix 2) accessed via a link sent to each participant by email. In addition, at the start of each interview, we asked participants whether they had read the participant information sheet (Multimedia Appendix 1) and whether they had any questions (which we answered if they did). We also asked them to confirm whether they were comfortable with the interview being recorded (refer to the interview topic guide in Multimedia Appendix 3). Study data were stored securely in pseudonymized form. We removed any potential identifiers from interview transcripts before analysis. The moderators’ participation in this study was voluntary, and they were not compensated for their participation.

## Results

### Participants

We interviewed all 6 people with a role in moderating the forum. The moderators employed by HealthUnlocked or ALUK had different job roles, with moderating the asthma OHC being only a part of their job. The patient ambassador, an experienced OHC user and patient with asthma, undertook the role of a moderator on a voluntary basis outside of her regular part-time employment. Table 1 presents a summary of the participants’ demographics and background characteristics.

**Table 1:** Details of the roles, background, and demographic characteristics of the Asthma + Lung UK (ALUK) asthma online health community moderators who participated in this qualitative interview study (N=6).

Characteristics			Moderators, n (%)
**Role**			
	Customer support manager employed by HealthUnlocked	1 (17)	
	Specialist respiratory nurse employed by ALUK	4 (67)	
	Patient ambassador (voluntary role)	1 (17)	
**Mode of work**			
	Part time	4 (67)	
	Full time	2 (33)	
**Experience (y)^a^**			
	<1	1 (17)	
	1-2	3 (50)	
	>2	2 (33)	
**Sex**			
	Female	6 (100)	
**Age group (y)**			
	30-39	2 (33)	
	40-49	3 (50)	
	60-69	1 (17)	
**Ethnicity**			
	Mixed, White British/Black African	1 (17)	
	White/White British	5 (83)	

^a^Years of experience at time of the interview.

The asthma OHC had approximately 1800 active members per month over a 12-month period (March 2022 to February 2023), including the period when the interviews were conducted (mean 1778, SD 69 active members), with approximately 90 new members joining each month (mean 94, SD 12 new members) and approximately 750 new posts per month (including threads and replies; mean 753, SD 155 new posts; Multimedia Appendix 4).

### Themes

**Figure 1:**
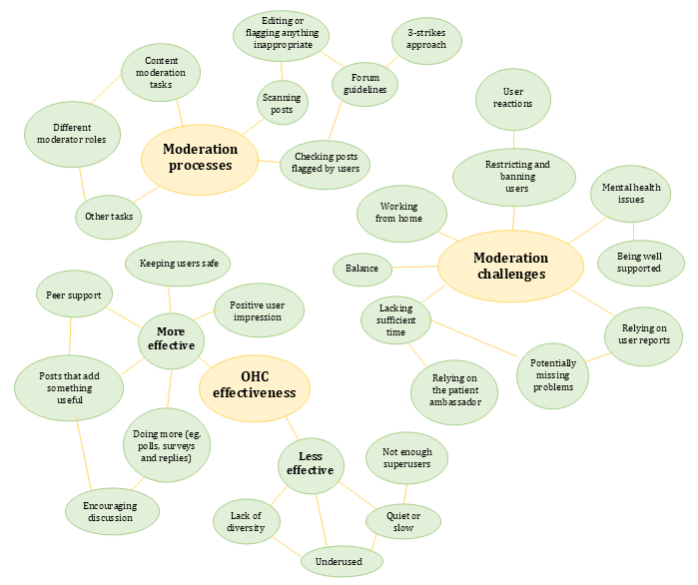
Overview of the 3 themes and associated subthemes derived from thematic content analysis of qualitative interviews of moderators of the Asthma + Lung UK asthma online health community (OHC).

From the data analysis, we generated three main themes: (1) moderation processes, (2) challenges to effective moderation, and (3) OHC effectiveness. Figure 1 provides an overview of the themes and associated subthemes.

#### Theme 1: Moderation Processes

##### Moderation Tasks According to Job Roles

Content moderation was the main task according to all moderators interviewed. This was described as checking posts for unsafe or inappropriate content and removing spam. These processes differed between the job roles (Table 2). In fact, the customer support manager only reviewed posts that were automatically flagged (using basic algorithms to detect spam and certain keywords) or reported by users:

We also have, for automatic spam...so the system picks up on those. What that does, it flags them, but it’s my job...there’s still a human that has to approve whether or not I agree with the automated system, so I still have to review it and say, “Okay, that should have been flagged”, or “No, that’s a false positive.”

Participant 2

My day-to-day tasks basically involve dealing with any reports that come in from the community...if they post something that offends or upsets somebody...it’s my job to investigate, either delete the post or, if the post has anything that might be triggering to some people, that falls into the guidelines, then I would make it community-only or put a trigger warning.

Participant 2

**Table 2:** Details of the different types of tasks involved in moderating the Asthma + Lung UK (ALUK) asthma online health community, according to the moderators’ different job roles.

Task			Customer support manager (HealthUnlocked)		ALUK nurses		Patient ambassador (ALUK team)
**Interaction with forum users**							
	Provide technical support to users	✓					
	Reply to posts and answer questions			✓		✓	
	Respond to unanswered posts			✓		✓	
	Welcome new users			✓		✓	
	Signpost to resources and share information			✓		✓	
	Share personal experience (as a patient)					✓	
	Encourage discussion			✓		✓	
	Post polls or “hot topics” for discussion			✓			
**Content moderation and resolution**							
	Apply forum guidelines as a framework for moderation	✓		✓		✓	
	Review posts automatically flagged by HealthUnlocked system	✓					
	Review posts flagged by users	✓		✓			
	Review posts flagged by the ambassador			✓			
	Scan or read all new posts			✓		✓	
	Edit or delete inappropriate posts	✓		✓		✓	
	Warn users posting inappropriate content	✓		✓		✓	
	Restrict or ban users who do not respond to warnings	✓		✓			
	Discuss concerns with other moderators or manager	✓		✓		✓	
	Out-of-hours cover					✓	
**Other**							
	Increase awareness of the forum on other channels			✓			

The ALUK nurses and patient ambassador described monitoring all newly posted content (new threads and replies, ie, those posted in the intervening time since they last looked at the OHC platform) in addition to posts reported by users:

I check to see if there’s been any reports of abuse. The members can report if they’ve seen a post that they think goes against the forum guidelines, so I respond to those. I can’t say I read every single post because I just wouldn’t be able to, there’s too much! So I kind of scan over it and have a look and see if there’s anything of concern.

Participant 5

I might just check in every so often, just scan through and look at the posts, click in, read, see if there is anything new...I scan to see if there is anything new, see if there is anything I want to reply to, anything I need to jump on immediately or anything I want to share with the nurse moderators.

Participant 1

Of note, the patient ambassador was the only moderator checking new content on the forum outside of normal office hours:

They [the ALUK nurse moderators] work with us in the UK and have other jobs so they’re not around at the weekends. I might be around at the weekends or on holiday, so I can jump in there and potentially get on top of things before they spiral out of control.

Participant 1

##### Interaction With Users According to Job Roles

Apart from providing technical support to users and contacting them (by private message) regarding inappropriate posts, the customer support manager did not have direct contact or interaction with users:

I try not to comment in the community, inside the actual community posts...I would never jump in into a post designated about the health issue itself.

Participant 2

In contrast, part of the role of the ALUK nurses and the patient ambassador was to interact with users, responding to posts to answer questions; signpost users to useful resources, including the ALUK website and helpline; as well as posting polls and surveys to generate discussion. They also highlighted the importance of welcoming new users and making sure that users receive a response to their posts, if not already answered by other users:

So we’ll firstly deal with any of the issues that they’ve raised as a concern. If we then need to reach out to individual forum members and say things like...we’re a bit worried about the advice that you’re giving, can we have a chat about it or, are you okay because you seem to be in distress? So, we’ll deal with those things and then we will have a look through all of the chats that are going on. If there’s any value for us to add then we’ll add in a comment here or there. If there’s any posts that no one has answered we might put a post in, just to support and encourage participation. Make people feel like they’re not being ignored. We might run a poll. We might share something of interest.

Participant 3

Also, if people are relatively new and they’ve asked a question—especially if no-one else has answered it—I’ll try to jump in. If it’s not something I’ve got any experience of but no-one’s answered, I’ll soon try to jump in there anyway and try to get things going even if it’s just to say, “I don’t know much about this, but hi and welcome and hopefully someone else will be able to answer that” or “here are some links”, or just general information.

Participant 1

##### Moderating Forum Guidelines

In assessing whether a post or private message (ie, a message sent between users, not published on the OHC and only seen by moderators if reported by a user) was against the rules and when contacting users, the moderators mentioned that they often referred to a published set of community guidelines, which users agreed to abide by when they signed up to the OHC:

If I get an inappropriate post, I review it to make sure what part of the terms and conditions it’s breaking, then I’ll delete the post and then I’ll send a warning to the member, via e-mail or chat,...and we send them the terms again just so that they’re aware, in case they didn’t know.

Participant 2

One of the moderators emphasized the importance of the moderation process being a 2-way conversation:

It’s not an automatic thing. We really try and engage with people before giving out warnings or putting in restrictions. I might say, “look, I’ve deleted this thread because it’s not in the spirit of the site” or “it’s against our guidelines” or whatever. “I know that might be a bit difficult for you, so do you want to have a chat about it?”

Participant 3

However, the moderators noted that users were not always responsive to their warnings and described using a “three strikes” approach:

A couple of times people have just ignored us and just carried on doing it. This used to happen more so when I first started it, and it’s sort of three strikes and you’re out! If they carry on and they do it three times we can just restrict them from the forum completely.

Participant 5

If a post did not warrant deletion, for example, posts containing dubious (but not dangerous) advice or recommendations, the moderators reported that they would edit a post to add a warning and signposts to appropriate resources:

I made the decision to leave the post up and edit it with a warning...I tend to do that if it’s been up for a few days and there’s been a lot of replies and quite often the replies are sensible, saying what I would have said in my warning...If it’s really bad I’ll just delete it, take the whole thing down.

Participant 5

All the moderators commented on the nature of the inappropriate content and the type of issues that required their intervention. These are summarized in Textbox 1.

**Details of the different types of content posted on the Asthma + Lung UK (ALUK) asthma online health community (OHC) that are inappropriate or contrary to OHC guidelines and therefore require intervention, as reported by the OHC moderators.**

**Spam issues**

- Selling products
- Links to research (without permission)

**Unpleasant language or behavior issues**

- Being rude or aggressive to another user
- Using foul language
- Hostile debate

**Advice or recommendations issues**

- Dubious or unhelpful advice
- Promoting alternative remedies
- Against taking medication or vaccines
- Describing unusual medication regimes
- Misinformation
- Contrary to ALUK guidelines or advice

**Potentially upsetting or triggering issues**

- Description of harrowing asthma attack
- Mental health issues
- Self-harm or suicidal thoughts

#### Theme 2: Challenges to Effective Moderation

##### Restricting or Banning Users

An aspect of the role that several moderators reportedly found challenging was having to restrict or ban users and dealing with their sometimes-unpleasant reactions to being sanctioned:

You don’t want to ban anyone, because you want everybody who needs access to the community to use it, but sometimes you have some members who just aren’t helping the flow of the community at all and they’re just upsetting everyone, so it’s not worth it, but sometimes they can be a bit unpleasant on e-mail [in response to being contacted].

Participant 2

I hate it when people...if they have put something which is unsafe and they’re really angry about it...trying to respond and try and be nice to them, but it’s hard—it’s challenging.

Participant 4

##### Users’ Mental Health Issues

Posts regarding users’ mental health were also an issue that all moderators described as being difficult to deal with, although they noted feeling supported, both within the moderation team and by their management. The ALUK nurses and the patient ambassador described a separate “moderator’s forum” where they could discuss and share issues among themselves. The customer support manager referred to a weekly “trust and safety committee,” where she could discuss more serious moderation cases:

I get very worried about mental health posts on the forum...I find it really hard because you’re on a forum and you don’t really know what’s happening—are they okay? I always worry if they’re going to be okay.

Participant 5

##### Balancing Moderation With Users’ Freedom of Expression

Another challenge perceived by the moderators was establishing a balance between allowing users to freely express themselves on the forum and post about their experiences while maintaining the community guidelines:

It’s about the balance between not letting things run away and get unhelpful or hostile, but letting people have a discussion. That’s the thing I find most challenging. Being there but not being too in their face about it.

Participant 1

We have to strike that balance between keeping everyone safe but not imposing our views in a way that might seem censorious or uncomfortable for people.

Participant 3

##### Time Constraints and Workload

The ALUK nurses commented that the lack of time was a key challenge in effectively moderating the OHC, resulting in a heavy reliance on the patient ambassador, especially during out-of-office hours. Notably, the ambassador, although a volunteer, described spending more time on the OHC than the individual nurses, which was around 2 to 5 hours per week:

So there’s an hour allocated to HealthUnlocked by us Monday to Friday every week. Sometimes it doesn’t work that way, if someone’s got to go off to another meeting or the helpline’s gone crazy on the phones, that hour doesn’t get the time. But it’s supposed to have five hours of nurse time Monday to Friday every week, and then the ambassador picks up all the other stuff.

Participant 5

The patient ambassador noted that the amount of time she was spending on moderating the OHC was “manageable.” She mentioned that there was previously a second patient ambassador a few years ago when the OHC was busier:

A few years ago I think it would have been more of a job. I think actually the reason that the other ambassador left is because it was more of a job and she didn’t have time for it. Now I think it probably wouldn’t be a huge amount of work for her especially with someone else.

Participant 1

The nurses, in particular, felt that needing to read or scan through every post was a very time-consuming activity and were concerned about not knowing whether someone else in the team had already looked at the posts:

So, we [are] either doubling up the workload or not doing it, but I always try and look for at least the last 24 hours but, then again, as well as looking through the replies—and people can reply to posts from ages ago if they want to—you might not always see it.

Participant 4

Yeah, stuff can get missed. Yeah, definitely. Because I think the only way stuff wouldn’t get missed is if we had someone moderating on there all the time, and it’s just not possible at the moment. We don’t have the staff or the capacity and I’ve only got one ambassador.

Participant 5

The reliance on user reports to flag potential problems was also noted, with one moderator commenting that it would be helpful if users would use the report button more:

If members don’t report a post or a comment that’s inappropriate or breaking the rules, a lot of the times I don’t see it, so there could be posts that haven’t been viewed that should have...I do rely heavily on members reporting, and if they don’t, then there is a chance you miss some things.

Participant 2

##### Working From Home

The final challenge mentioned by a member of the nurse moderator team was working from home and missing the benefits of working in proximity to her colleagues that were available before the COVID-19 pandemic:

We’re all on working from home contracts so we’re all working in isolation. But we are fighting to get back at least one or two days a month, [to] the office where we can all work together. Because, you know, we learn so much more from each other and can be I think more effective and creative when we work together and lots of learning can take place.

Participant 6

#### Theme 3: OHC Effectiveness

##### Keeping Users Safe

Several moderators talked about the satisfaction they got from knowing that they kept users and the community safe by effectively moderating. In particular, the patient ambassador commented that when she first joined the OHC (before it was managed and hosted by HealthUnlocked), moderators were much less present, and it consequently “felt almost unsafe”:

Because of how active our moderation is, members know that if someone were to break the rules, it would get sorted fairly quickly, which gives people peace of mind and makes them comfortable, because no-one wants to join a community where there’s trolls everywhere and nobody’s actually moderating.

Participant 2

I think making everybody feel safe. Making people know that unacceptable behaviour’s not tolerated. That that will always be stepped on and managed.

Participant 3

##### Positive User Impression

The impression among the moderators was that they were generally appreciated by the users for their involvement in the community:

They might feel happy and reassured that there’s a nurse there answering their posts, and that they can get an answer to a question if they want to.

Participant 4

They perceived the users, for the most part, in a very positive way, describing them as “pretty civilised,” “very polite and well behaved,” and “very pleasant and nice,” with a nurse noting that she had never received any “negative feedback” from users while another mentioned receiving “nice feedback.” There was a general feeling of the OHC being a pleasant environment and the moderators enjoying their involvement in it:

I love hearing what people have to say. I like witnessing the lived experience of people with often quite severe asthma...I like being able to answer problems or give perspective but really, I enjoy observing the peer support and the sense of community that HealthUnlocked brings to them.

Participant 3

##### What Makes OHC Engagement Effective

In terms of why people used the OHC and what they gained from it, the moderators talked about the benefits of peer support, sharing experiences, and how useful people found it:

They’ll offer real life solutions, and they’ll offer little tricks to get what you need...they can just offer that level of understanding and support that only someone who has that lived experience can offer really and that’s a lot more meaningful to people when they feel alone and isolated, than speaking to a healthcare professional.

Participant 3

I can see that the people that are on our forum really get a lot of benefit from it...And we’ve got a lot of members, we’ve got nineteen and a half thousand members. I can’t say all of those are active. So it clearly does help a lot of people.

Participant 5

I think it’s people sharing their personal stories that people really like and their experiences.

Participant 4

The most useful posts or conversations were felt to be those where users provided new information, ideas, or constructive advice and where they had read the question properly such that the replies were relevant:

I think it’s effective if people come in and give them information or things to think about that they haven’t considered or they weren’t looking for because they didn’t realise that they wanted it...if people can offer constructive advice that actually helps them work it out, that kind of problem. It’s not so effective if people haven’t really read the question. I think people really reading and understanding the question and then responding and adding something to it.

Participant 1

Several moderators highlighted their own role in improving the forum and making it a reliable, friendly environment for users:

I like the fact that as a moderator I feel I could be more productive for the forum and make sure it is friendly, helpful and actually a useful place to be.

Participant 1

I do like the fact that we’re dealing with posts that are reported on by other members, so it shows that members know that they can rely on us.

Participant 2

...keeping people safe and signposting them to helpful websites or, you know, the relevant help that they may need. And protecting others from bad advice I think as a clinician is what we should be doing.

Participant 6

The importance of the role played by the patient ambassador in providing support was recognized by the ALUK nurses. They highlighted the advantages of being a frequent and long-standing user of the OHC as well as a relatable peer and a trusted source of information:

...they’re the eyes and ears of what’s happening on the site, and they do a phenomenal job and what’s nice about them is that they really encourage that peer support.

Participant 3

They [the users] might not want to speak to us nurses because they might be thinking—they might have a negative view of all health professionals at that point and that’s where the health ambassadors can be really, really useful.

Participant 3

Increasing engagement with and awareness of the OHC among potential users was seen as an avenue by which community effectiveness could be improved:

The value for the people that use the forum seems to be immense but relatively few people use it.

Participant 3

What I quite often do is if I’m speaking to somebody on the phone and I say, you would really love this forum. Go on there.

Participant 3

##### Importance of OHC Superusers

Two moderators highlighted the importance of well-established or particularly active users (“superusers”) in responding to posts and driving activity on the OHC:

It was talking about superusers and how they drive things. I thought maybe that’s why, we haven’t got enough superusers! I’ve noticed just even as a user, there are people who post quite a lot and we don’t really have those now.

Participant 1

...if someone new makes a post, the people who’ve been on the community longer definitely are quick to respond and to offer any assistance or advice.

Participant 2

##### Lack of Diversity Among Users

One of the nurses commented on diversity among the OHC users being a challenge, in particular, the lack of younger users and the impact of the OHC format on user recruitment:

It’s challenging to get a vibrant community going that represents lots of different ages and ethnicities.

Participant 3

I just note when I look at the demographic of HealthUnlocked that doesn’t really seem to be changing. Is that we haven’t really got anybody below the age of 30 and there must be a reason for that, but I don’t know what that reason is. I suspect it’s just because it’s not a format that appeals to young people but that’s me guessing.

Participant 3

##### How to Improve OHC Effectiveness

Several moderators talked about the need for them to be more involved in the forum in a positive way, doing more to generate activity and encourage discussion on the OHC and adding value and content, as opposed to just focusing on “the negative side” of moderating, that is, enforcing the community guidelines:

I think it probably is too little, and it’s only when you sit down and have these kind of conversations and I think that, as a moderator...I focus too much on trying to keep it all safe—making sure nobody’s putting anything negative on there—rather than trying to interact and probably be a little bit more present in a positive way in the forum.

Participant 4

I sometimes think, oh, should we be doing more? Should we be doing more polls? Should we be trying to make it a bit more whizzy and interactive but I think we would be doing that more for ourselves than we’d be doing it for the patients because I think actually, they’re quite happy.

Participant 3

One participant highlighted the issue of whether the users wanted the moderators to be more involved or whether they might consider the nurses to be overstepping in what was essentially a “peer-to-peer forum”:

If activity’s gone down then I’ll put some posts out just to try and boost the interest. It’s really hard because some [users] of the forum seem to really like us getting involved and others, I feel like they don’t.

Participant 5

One of the nurses did note that when they posted polls, these were well received by the users:

I think, where I’ve noticed—where things get lots of interaction—are polls. People quite like a poll, and the questions where they can share their experiences...I think it’s people sharing their personal stories that people really like.

Participant 4

## Discussion

### Principal Findings

The findings of this study illustrate the various roles and responsibilities involved in moderating an OHC. In this charity-hosted asthma OHC, there were no significant issues with inappropriate user behavior or content, and the moderation process worked well. However, challenges were identified, including having to restrict or ban users, dealing with users’ mental health issues, balancing moderation with users’ freedom of expression, managing time constraints and workload, and working from home. Moderators’ views on what makes the OHC effective highlighted areas that could be targeted to optimize the safety and effectiveness of the forum, including generating more OHC activity, encouraging discussion, and raising awareness of the OHC. On the basis of these results, we derived a set of recommendations that were grouped under the 3 themes identified earlier (Textbox 2). This study shows that OHC moderators typically operate as a cohesive unit within their organization or community without engaging with moderators from other OHCs or having access to external professional moderation training opportunities.

**Recommendations for optimizing the safety and effectiveness of the Asthma + Lung UK (ALUK) asthma online health community (OHC), derived from thematic analysis of qualitative interviews with the moderators of this OHC.**

**Theme 1: moderation processes**

- Introduce training and a resource on the moderators’ forum (used by nurse moderators and the patient ambassador) to remind the team about the automated process and how it works and to ensure that everyone is familiar with the functionality to assist in moderating, such as searching posts and selecting unanswered posts
- Recruit another patient ambassador

**Theme 2: challenges to effective moderation**

- Explore whether there is a mechanism for posts to be marked as “read” by moderators (in a similar way to emails) to facilitate shared moderation and reduce the risk of duplication of effort or missing potential problems
- Explore the possibility of increasing the number of hours allocated to the moderation team
- Explore how time allocated for moderation is distributed across the moderation team during office hours versus out-of-office hours
- Ensure that all moderators are aware of the strategy adopted by the hosting platform in dealing with self-harm, suicide, and mental health issues
- Explore the possibility of the ALUK moderator team working together in the office on a regular basis to enhance peer support and team cohesiveness, for example, on a monthly basis

**Theme 3: OHC effectiveness**

- Increase promotion of the asthma OHC on charity social media and other channels where possible, including nurses promoting the OHC to users of the charity helpline
- Encourage discussion through some kind of regular feature, such as a monthly poll or “hot topic” generated by the nurses
- Post a survey asking users about how they use the OHC; what they would like to see on the OHC; and whether they would like the health care professional moderators to be more involved, including specific questions exploring whether the users would see wider moderator participation as destroying the peer-to-peer nature or helpful

This study also highlights the vital role that knowledgeable and experienced moderators play in the ALUK asthma OHC. The range of different job roles involved in moderating brought an important range of skills and perspectives. While all the moderators were involved in keeping patients safe by removing inappropriate and unwanted content, each role added further value. The customer support manager gave practical support to users through technical assistance. The patient ambassador added a wealth of lived experience as a patient with asthma, connecting with users as a knowledgeable peer in a friendly and relatable way while maintaining the ALUK recommendations. The patient ambassador made a point of welcoming new users and providing answers to questions that had not been addressed by other users, an important contribution to the community *feel* and the forum’s usefulness to users. The nurse moderators brought their specialist and up-to-date knowledge of health practice and ALUK recommendations, warning users about any potentially risky advice or practices and giving validity to the forum as a source of safe information. Furthermore, they contributed directly to the forum, posting polls to generate discussion and ensuring that important posts were “pinned” on the home page where they would be most visible to users.

### Comparison With Existing Literature

Few studies of OHCs have focused on exploring the role of moderators and moderation processes [13-18], with none of these being specific to asthma or even to lung conditions in general. While moderators may be important for the vibrancy of OHCs, improving patient outcomes and making members feel safe, investing in hiring them (especially those with clinical backgrounds), and motivating them to participate voluntarily can be difficult [14,16]. In a study of 6 different OHCs within the WebMD platform, Huh et al [15] recognized the distinct roles played by health professionals and peer patients, highlighting the beneficial “synergistic efforts” of these groups in creating and sustaining vibrant communities. Furthermore, they mentioned staff moderators, who were nonclinical staff and worked across multiple OHCs but had a different role from the customer support manager in our study, as they also replied directly to patient posts.

Our findings align with those of Deng et al [18] and Skousen et al [17] in recognizing the vital role of moderators in OHCs, including the importance of volunteer patient moderators. In a study exploring the perspectives of moderators of the Togetherall peer support platform, an OHC for mental health, Deng et al [18] highlighted the value of insights offered by moderators in improving the quality of online forums, addressing potential challenges, such as managing emotionally triggering issues, users being hostile or willfully breaking community rules, gauging the right level of moderation when editing users’ posts, and overcoming low user engagement. The authors concluded that moderators play a potentially critical role in maximizing the safety and chances of the beneficial impact of an online peer support community and highlighted the importance of moderators being well trained and having relevant clinical expertise [18].

A study by Skousen et al [17] described how moderators of the Connect OHC, a patient community sponsored by the Mayo Clinic, identified emerging peer leaders and invited them to assist with moderating the community. Peer leaders are similar to the patient ambassadors described in our asthma OHC. These “mentors” volunteer their time and are recognized as having significant advantages, reducing the workload of professional moderators and holding the unique perspective of “shared experience” (ie, having themselves experienced the issues likely to be raised by peers). The study highlighted the importance of training for volunteer mentors and guidance from professional moderators [17]. Patient moderators of online support groups were the focus of the study conducted by Coulson and Shaw [13]. They highlighted several challenges, including the time commitment required for moderation, dealing with trolls and “nasty emails,” and striking the right balance between their nurturing role and enforcing the rules. Patient moderators could, over time, develop the personal skills and qualities “necessary” to undertake the role, but the need for appropriate upfront training so that they might be better equipped from the outset was also highlighted. In our study, the patient ambassador, a long-time user of the asthma OHC, described how she had become more confident in her role over time but would still seek advice from the ALUK nurses if unsure. Although there was only 1 patient ambassador at the time of this study, superusers may also offer support in this role. Their contributions to moderation, such as promptly reporting posts, responding to risk-indicating or inappropriate posts, and correcting posted information, were documented in a previous study on the same OHC [24].

Training and ongoing updates are important for both professional and patient moderators. For the specialist respiratory nurses moderating the ALUK asthma OHC, this is only a part of their role, their main job being the ALUK helpline. As such, it is a necessary part of their role to stay up to date with new therapies and treatments as well as ALUK guidelines. We did not specifically ask about training in our interviews, but this is an area that could be explored further in the future.

### Interpretation of Findings Using the Framework of the Behavior Change Wheel

In an interview study with moderators of a young people’s OHC, Perowne and Gutman [25] used the behavior change wheel, a framework developed to characterize behavior change interventions [26], to identify barriers and enablers to effective moderation. Most of the enablers and barriers identified are reflected in our findings from this study, as summarized in Table 3.

**Table 3:** Details of enablers and barriers to effective online health community (OHC) moderation identified in a study by Perowne and Gutman [25] and also found in this qualitative study examining moderation of the Asthma + Lung UK asthma OHC.

Enablers and barriers			How these are related to this study, as evidenced by the OHC moderators
**Enablers**			
	Knowledge of the issue being discussed	Moderators highlighted the importance of understanding asthma and its complications.	
	Knowledge of the OHC guidelines	OHC guidelines were referred to by all moderators in describing their roles and the processes involved in moderation.	
	Being empathetic in communications	Moderators mentioned the importance of making users feel supported and not alienated.	
	Skills developed through the experience of moderating	The skills were reflected by the patient ambassador, who said she was still learning the balance between moderating and overstepping.	
	Attention and focus to avoid missing things	Moderators mentioned the importance of careful scanning to avoid missing problematic posts.	
	Judgment in balancing different needs	Moderators described the need to strike a balance between moderating and letting users have a discussion.	
	Confidence increasing through practice	This was described by the patient ambassador.	
	Aiming to create a safe and supportive space	Moderators mentioned the importance of making users feel safe as part of their role.	
	Wanting to moderate	Moderators talked about their desire to help others.	
	Discussion with other moderators before taking action	This was demonstrated by the patient ambassador checking in with the nurse moderators if unsure and the moderators having their own “moderator’s forum” on which they shared and discussed issues.	
	Influence of the community users	Moderators were assisted by users flagging posts of concern.	
**Barriers**			
	Emotional exhaustion	This was reflected in moderators worrying about posts regarding users’ mental health issues.	
	Working with partial information about users	Moderators described concern over not having a full picture when users posted about their mental health and not being able to find out whether they were okay (after they posted).	
	Lack of access to timely support	This was described by the patient ambassador when moderating outside of office hours and not being able to contact the nurse moderators.	
	Influence of the community users	Moderators described users being upset about their posts being edited or deleted or holding challenging views.	

Perowne and Gutman [25] additionally identified behavior change techniques that could potentially address barriers to effective moderation, which are partly reflected in our recommendations (Textbox 2). Specifically, these include (1) reducing negative emotions (we recommend training and awareness for the moderators in dealing with self-harm, suicide, and mental health issues), (2) restructuring the physical and social environment (we recommend enabling regular face-to-face team working opportunities), and (3) social support (we recommend increased team working opportunities, exploring how moderation work is distributed across the team, particularly for out-of-hours cover, and recruiting further patient ambassadors). The barrier of working with partial information about users is difficult to address, given the nature of an online forum and its limitations, that is, not being able to obtain further information about a user’s well-being unless they post publicly or reply to a private message, along with the requirement for OHC users not to disclose personal information. However, it has been previously noted that the anonymity of an OHC may be helpful to users in opening up and sharing more information [27].

### Limitations

The main limitation of our study is the small sample size (involving 6 moderators, all of the same sex, with only 1 patient ambassador and 1 customer support manager) and the involvement of moderators from a single OHC. Hence, our findings might not be generalizable. However, a strength of the study is that all current moderators of a discrete large and active OHC were interviewed in depth to explore their experiences in detail, giving moderators time and space to fully express their views. Therefore, a baseline overview of the moderation processes and challenges was obtained, enabling analysis and planning of future actions to optimize moderation and, eventually, OHC safety and effectiveness. Our findings are relevant to other OHCs designed for peer support among patients with long-term conditions, which could benefit from the suggested recommendations.

### Implications for OHC Moderation and Research

OHC moderators tend to work as a group within an organization or community without interaction with moderators of other OHCs or opportunities for external professional moderation training. This situation prompts the following critical question: Could the implementation of a continuous professional development framework for OHC moderators be a beneficial and transformative approach to enhance the quality of moderation and support for OHC users? Investigating the moderation practices and policies of established OHCs can offer valuable insights into the potential advantages of continuous professional development for moderators and how it aligns with the evolving needs of these dynamic communities. The scope of potential benefits to patients and health services from effective online health support communities is significant, including improved self-management and health literacy, as well as reduced dependence on primary care services. As such, it is of vital importance that these communities are properly moderated, providing safe advice and support to their users.

Our analysis highlighted issues and challenges to the moderation process and areas where the effectiveness of users’ OHC engagement could potentially be optimized. Future research is needed to verify whether the set of recommendations proposed in this study is implementable and effective in improving moderation, both from the moderators’ and users’ perspectives. The recommendations might inform national and international policy, attempting to enhance the safety of patients’ engagement with OHCs. Findings will also assist OHC moderators in ensuring effective interactions among OHC users.

## Appendix

Multimedia Appendix 1 Participant information sheet for moderators invited to participate in a qualitative interview study examining moderation of the Asthma + Lung UK asthma online health community.

Multimedia Appendix 2 Consent form for moderators participating in a qualitative interview study examining moderation of the Asthma + Lung UK asthma online health community.

Multimedia Appendix 3 Interview topic guide for qualitative interviews with moderators of the Asthma + Lung UK asthma online health community.

Multimedia Appendix 4 Details of user engagement, in terms of membership and content, for the Asthma + Lung UK asthma online health community over the 12-month period from March 2022 to February 2023.

## Abbreviations

ALUK: Asthma + Lung UK
OHC: online health community

## Data Availability

The datasets generated and analyzed during this study are available from the corresponding author on reasonable request.
