# Supplementary material for: Moderators’ Experiences of the Safety and Effectiveness of Patient Engagement in an Asthma Online Health Community: Exploratory Qualitative Interview Study
Source: J Med Internet Res. 2025 Apr 25;27:e58167. doi: 10.2196/58167 (PMC12064959; doi:10.2196/58167)
Supplement: Multimedia Appendix 1 [file jmir_v27i1e58167_app1.pdf]

Information Sheet for Participants

How optimising the safety and effectiveness of patients' engagement in the Asthma+Lung UK Online Health Community (OHC) can inform the OHC moderation process

Summary

AD HOC is a programme of research studies investigating whether the promotion of engagement with the Asthma+Lung UK online health community (OHC) in primary care improves patients' asthma control and self-management.

This information sheet is about the part of the programme optimising the OHC moderation process through a set of recommendations, developed from qualitative and network science analyses of discussion threads in existing datasets of the Asthma UK and British Lung Foundation OHCs.

We would like to hear about your moderator's experiences before and after these recommendations are implemented, and your views on the recommendations themselves.

Why have I been invited to participate?

As a moderator of the Asthma+Lung UK OHC, you are the target audience for the set of recommendations we will develop. We would like to measure your experiences as an OHC moderator before and after these recommendations are implemented, to help us understand whether they are effective and easy to use.

What will I be asked to do?

If you decide to take part in this research, we will first ask you to sign a consent form (a copy is attached). We will invite you to take part in a one-to-one interview with one of the researchers from the AD HOC team, before and 6 months after the recommendations are implemented. We will ask you about your experiences of moderating the Asthma+Lung UK OHC, including any challenges you have faced in improving user engagement and ensuring users' safety. At the second interview, we will specifically ask you about the new recommendations and how these have affected your experiences as a moderator.

The interviews will be undertaken in person, if convenient, or online otherwise, in a private location where you will not be overheard. Data from the Interviews will be collected by note writing and digital audiotaping.

What will happen to the data collected?

The interview recordings will be transcribed (typed up). The interview notes, recordings and transcripts will be anonymised and stored, along with personal contact details (telephone number/email), in compliance with all GDPR regulations. Data will be processed in accordance with GDPR, and will be used and accessed only by the AD HOC research team. Any personal identifiers used during the interview will be removed at transcription (e.g. references that would allow an individual to be identified). Quotes used in any publications deriving from this research will contain no identifiable information that could connect them back to a particular individual.

Possible benefits and disadvantages of taking part

By taking part you will help us to understand the experiences of the OHC moderators and the impact of the new recommendations on your experiences. This will help optimise the safety and effectiveness of OHCs, with the aim of improving users' engagement and experience.

There will be no disadvantages for you, whether or not you choose to take part. Interviews will last approximately between 20 minutes and 1 hour and will be arranged at times convenient to you.

Why we want to do this research

- Around 4.3 million adults in the UK are affected by asthma, with up to one third of these experiencing poor control of their asthma symptoms.
- This leads to a large number of GP consultations, A&E visits, and hospitalisations.
- Online health communities are increasingly used as a source of lay health advice.
- There is some evidence that interacting with peers online in trusted OHCs could improve self-management and health.
- We want to find out whether promoting engagement with the Asthma+Lung UK OHC in primary care helps patients to control their asthma symptoms better.
- Optimising user safety and the effectiveness of user engagement with the OHC is a first step before we can investigate its impact on patients.

What happens next?

It is up to you to decide whether or not to take part. If you do decide to take part you will be given this information sheet to keep and be asked to sign a consent form. You are free to withdraw at any point and do not have to provide a reason for this.

Please read the information carefully before you decide to take part; this will tell you why the research is being done and what you will be asked to do if you take part. Please ask if there is anything that is not clear or if you would like more information.

If you have any questions or concerns about the manner in which this research was conducted please, in the first instance, contact the researcher responsible for the study. If this is unsuccessful, or not appropriate, please contact the Secretary at the Queen Mary Ethics of Research Committee, Room W104, Queen's Building, Mile End Campus, Mile End Road, London or research-ethics@qmul.ac.uk.

Please read Queen Mary's privacy notice for research participants for important information about your personal data and your rights in this respect.

This is found at: http://www.arcs.qmul.ac.uk/media/arcs/policyzone/Privacy-Notice-for-Research-Participants.pdf

Contacts details

If you have any questions about the study please contact us by email: ADHOC@qmul.ac.uk

Principal Investigator

Dr. Anna De Simoni
Wolfson Institute of Population Health, Queen Mary University of London
Email: a.desimoni@qmul.ac.uk
Voicemail: 020 7882 2520

Thank you for taking the time to read this information sheet
