# Supplementary material for: Moderators’ Experiences of the Safety and Effectiveness of Patient Engagement in an Asthma Online Health Community: Exploratory Qualitative Interview Study
Source: J Med Internet Res. 2025 Apr 25;27:e58167. doi: 10.2196/58167 (PMC12064959; doi:10.2196/58167)
Supplement: Multimedia Appendix 2 [file jmir_v27i1e58167_app2.pdf]

Consent Form

How optimising the safety and effectiveness of patients’ engagement in the Asthma+Lung UK Online Health Community (OHC) can inform the OHC moderation process

Please complete this form after you have read the Information Sheet (V1.0 18.08.2022) and/or listened to an explanation about the research.

Thank you for considering taking part in this research. A member of the research team must explain the project to you before you agree to take part.

Statement	Please initial box
1. I confirm that I have read the Participant Information Sheet dated [insert date] version [insert version] for the above study; or it has been read to me. I have had the opportunity to consider the information, ask questions and have had these answered satisfactorily.	
2. I understand that my participation is voluntary and that I am free to stop taking part in the study at any time without giving any reason and without my rights being affected.	
3. I understand that my data will be accessed by the research team.	
4. I understand that my data will be securely stored in the Wolfson Institute of Population Health and in accordance with the data protection guidelines of the Queen Mary University of London for 20 years in pseudonymised/non-identifiable form.	
5. I understand that I can access the information I have provided and request destruction of that information at any time prior to publication. I understand that following publication I will not be able to request withdrawal of the information I have provided.	
6. I agree to the interview being audio recorded.	
7. I understand that the researcher will not identify me in any publications and other study outputs using personal information obtained from this study.	
8. I understand that the information collected about me will be used to support other research in the future, and it may be shared in anonymised form with other researchers.	
9. I agree to take part in the above study.	

Participants should read Queen Mary's privacy notice for research participants which contains important information about your personal data and your rights in this respect. If you have any questions relating to data protection, please contact Data Protection Officer, Queens' Building, Mile End Road, London, E1 4NS or data-protection@qmul.ac.uk or 020 7882 7596.

_________________ ______________ _______________________________

Participant name Date Signature

___________________ ________________ __________________________________

Name of person taking consent Date Signature

I [insert Investigator Name] confirm that I have carefully explained the nature, demands and any foreseeable risks (where applicable) of the proposed research to the participant and provided a copy of this form.

Principal Investigator
Dr. Anna De Simoni
Email: a.desimoni@qmul.ac.uk
Voicemail: 020 7882 2520
