# Supplementary material for: Moderators’ Experiences of the Safety and Effectiveness of Patient Engagement in an Asthma Online Health Community: Exploratory Qualitative Interview Study
Source: J Med Internet Res. 2025 Apr 25;27:e58167. doi: 10.2196/58167 (PMC12064959; doi:10.2196/58167)
Supplement: Multimedia Appendix 3 [file jmir_v27i1e58167_app3.docx]

**Interview Topic Guide**

**Consent**

1. Who I am, what I am doing.
   1. My name is <name> and I’m from <institution>. Thank you so much for taking the time to talk with me today. I would like to talk to you about your experience with moderating the Asthma+Lung UK online health community, which I’ll refer to as ‘the OHC’. You should have already received an email with a Participant Information Sheet that contains detailed information about this study.
2. Privacy and confidentiality
   1. To clarify, your participation in this conversation is strictly confidential—we may quote some of your responses in a publishable paper, but your identity will never be published alongside those quotes. In other words, your responses may be published as quotes in a future paper, but no identifiers will link your username or identity to those quotes. Please feel free to be completely honest and open – this recording and our notes will only be used by the research team – your responses won’t be shared with your employer. You don’t have to answer any of the questions I ask, if you don’t want to. You are also free to stop the interview at any time, you just have to let me know.
3. Consent to interview and recording
   1. For the record, are you happy for me to record this conversation?
4. Participant questions
   1. Do you have any questions about this interview before we start?
5. Explanation of the open interview structure
   1. Just to let you know, there are no wrong answers to any of my questions. I’m interested to hear your stories and your experiences. This is your interview.

**Interview Questions**

1. Your role as a moderator
   1. How long have you been a moderator for the Asthma+Lung UK OHC?
   2. Could you tell me about what you do in your moderation work?
   3. How would you describe your workload as a moderator?
      1. Is this your only/main role or just one part of your job?
   4. Could you describe for me what a typical day of moderating the OHC involves for you?
   5. What’s the best thing about being a moderator/what do you like about it?
   6. Is there anything you don’t like or enjoy about this role?
2. The process of moderation
   1. Can you tell me about how the process of moderation works for the Asthma+Lung UK OHC?
      1. Is there an automated process that flags up posts containing particular words?
      2. Do you see posts that are flagged by users as potentially inappropriate?
      3. Is it language or behaviour or both that is flagged?
      4. What is the process for dealing with an inappropriate post?
      5. Do you see a sample of all the posts?
   2. Are there other aspects to the role beyond dealing with inappropriate posts?
      1. Do you initiate discussion threads or reply to posts?
      2. Does your role involve providing information to users?
3. What are the challenges/barriers to effective moderation?
   1. How do you think the user community feel about the moderators?
      1. Do they feel the moderators are useful/helpful to the community?
      2. Do they feel the moderators limit free discussion?
   2. Do the current processes always identify posts that should be flagged?
   3. What are the difficulties in moderating the community?
   4. What would make it easier to moderate the OHC effectively?
4. What makes a conversation on the OHC effective?
   1. What is the moderator’s role in helping to create effective conversations?
   2. What do you think about the level of moderator involvement? Too much/too little?
   3. What could the moderator do to further engage community users and make their experience of the community more effective?
5. Anything else…
