# Supplementary material for: Moderators’ Experiences of the Safety and Effectiveness of Patient Engagement in an Asthma Online Health Community: Exploratory Qualitative Interview Study
Source: J Med Internet Res. 2025 Apr 25;27:e58167. doi: 10.2196/58167 (PMC12064959; doi:10.2196/58167)
Supplement: Multimedia Appendix 4 [file jmir_v27i1e58167_app4.docx]

**Asthma forum monthly engagement metrics over a 12-month period**

| **Month** | **All members** | **Active members** | **New members** | **New content*** |
| --- | --- | --- | --- | --- |
| Mar-22 | 18,862 | 1,839 | 94 | 708 |
| Apr-22 | 18,934 | 1,827 | 77 | 962 |
| May-22 | 19,026 | 1,781 | 95 | 597 |
| Jun-22 | 19,104 | 1,689 | 83 | 643 |
| Jul-22 | 19,187 | 1,765 | 86 | 836 |
| Aug-22 | 19,248 | 1,688 | 72 | 653 |
| Sep-22 | 19,336 | 1,916 | 98 | 576 |
| Oct-22 | 19,434 | 1,792 | 101 | 689 |
| Nov-22 | 19,527 | 1,709 | 98 | 594 |
| Dec-22 | 19,635 | 1,728 | 114 | 823 |
| Jan-23 | 19,740 | 1,837 | 109 | 957 |
| Feb-23 | 19,834 | 1,768 | 101 | 994 |
| Mean | 19,322 | 1,778 | 94 | 753 |
| SD | 318 | 69 | 12 | 155 |

* Includes new threads and replies; SD, standard deviation; data provided by HealthUnlocked
